# Association Between the Patterns of Five Unhealthy Behaviors and Suicidal Behaviors Among Adolescents in Six Provinces of China

**DOI:** 10.3389/fpsyt.2022.901537

**Published:** 2022-07-05

**Authors:** Chunyu Guo, Yanni Xue, Zhengmei Xia, Yingying Cui, Jie Hu, Xuexue Huang, Yuhui Wan, Jun Fang, Shichen Zhang

**Affiliations:** ^1^Department of Toxicology, School of Public Health, Anhui Medical University, and Key Laboratory of Environmental Toxicology of Anhui Higher Education Institutes, Hefei, China; ^2^Department of Maternal, Child and Adolescent Health, School of Public Health, Anhui Medical University, and MOE Key Laboratory of Population Health Across Life Cycle/Anhui Provincial Key Laboratory of Population Health and Aristogenics, Hefei, China; ^3^Faculty of Pharmaceutical Science, Sojo University, Kumamoto, Japan; ^4^School of Public Health and Health Management, Anhui Medical College, Hefei, China

**Keywords:** behaviors, adolescent, latent class analysis, suicide ideation, suicide plan, suicide attempt

## Abstract

**Background:**

In adolescents, multiple unhealthy behaviors frequently occur together and are likely to be associated with suicidal behaviors (SBs), increasing the risk of suicide. This study aimed to clarify the potential patterns of unhealthy behaviors in Chinese adolescents and to examine the associations between the different patterns of unhealthy behaviors and SBs.

**Methods:**

A total of 22,628 middle school students were enrolled in this study. Self-reported unhealthy behaviors and SBs were investigated using questionnaires. Latent class analysis (LCA) was performed based on five unhealthy behaviors [smoking, alcohol use (AU), diet pill use (DPU), screen time (ST), and problematic mobile phone use (PMPU)]. Multivariate logistic regressions were used to examine associations between the different patterns of unhealthy behaviors and SBs.

**Results:**

Four subgroups of unhealthy behaviors were identified by LCA, including high-risk class (smoking/AU/DPU/PMPU/ST), moderate-risk class 1 (DPU/PMPU), moderate-risk class 2 (smoking/AU/ST), and low-risk class. Compared with the low-risk class, moderate-risk class 1, moderate-risk class 2, and high-risk class had higher risks of suicidal ideation, suicide plan, and suicide attempt.

**Conclusions:**

These findings suggested that patterns of unhealthy behaviors were related to SBs in Chinese adolescents. Accordingly, considerations of different classes of unhealthy behaviors may be essential for developing effective preventive programs.

## Introduction

Suicide is the fourth leading cause of death in adolescents around the world (1). In the general population, the single most important risk factor for suicide is a prior suicide attempt as one of the suicidal behaviors (SBs) (1). SBs, including suicidal ideation, suicide plan, and suicide attempt, are a sequence of behaviors that patients usually move from one to the other and eventually to completed suicide during the evolution of mental problems (2). Indeed, adolescents with SBs are more likely susceptible to completed suicide in later lifetime (3). Substantial published studies indicate that there is a high proportion of SBs in adolescents. In a Canadian study, 10.8% of adolescents exhibited suicidal ideation and 3.0% reported a suicide attempt (4). This problem seems more serious in the USA, with 17% of American teens having considered suicide, 14% having made suicide plan, and 8% having attempted suicide, according to the Centers for Disease Control and Prevention (5). In Chinese middle school students, the rate of suicidal ideation, suicide plan, and suicide attempt was 15.1, 7.2, and 3.5%, respectively (6).

Adolescence is a vital period for individuals to establish health behaviors as adolescents move into a life stage during which behaviors become more habitual (7). Simultaneously, many unhealthy behaviors, such as smoking, alcohol use (AU), diet pills use (DPU), problematic mobile phone use (PMPU), and screen time (ST), often develop during adolescence (8–11). Unhealthy behaviors are not only associated with poor healthy conditions of future life for adolescents, but also related to mental health problems that cannot be ignored for the healthy development of young people. For example, both cigarette smoking and alcohol drinking are associated with affective temperament and psychiatric distress, including anxiety and depression, and some suicidal ideation in adolescents (8, 12, 13). In a previous study, it was reported that affective temperament types were independently associated with lifetime suicide attempts (14). Furthermore, adolescents are more likely to misperceive their weight status, especially, girls often perceive that they are overweight even though their weights are normal (15). Perceived overweight is usually associated with the risk of using diet pills and skipping meals (9). Being obsessed with the use of diet pill may cause further depression, psychiatric disorders, and suicide (16, 17). In addition, electronic devices are popular among young people to spend daily time, which entails sedentary life style. In the USA, 43% of adolescents have overly long ST (18). And, teenagers spend an average of 7–8 h/day engaging in electronic devices in Canada and USA (11). Furthermore, it is known that subjects at risk for SB usually approach suicide through searching for information and news regarding self-harm and SBs on the Internet, especially among adolescents (19). Both high ST and PMPU have been reported to correlate with mental health problems, psychiatric disorders, and thought of suicide, which is due to increased sleep problems (20–23).

Under this circumstance, in this study, we focused on five unhealthy behaviors including smoking, AU, DPU, PMPU, and ST, and examined their potential relationship with SBs. Noteworthily, unhealthy behaviors usually do not appear separately, but tend to occur simultaneously and cluster together (24). In recent years, a few researchers have begun to identify the distinct classes of unhealthy behaviors using latent class analysis (LCA) (25). For instance, McFeeters et al. (26) examined the subclasses of stressful life events related to suicide, and Bernanke et al. (27) identified the relationship between mental health problems and SBs. However, there are few studies that pay attention directly to the associations between unhealthy behaviors and SBs based on LCA. It is meaningful to consider multiple factors/behaviors and their interactions for the prevention of suicide in adolescents. Thus, we hypothesized that some patterns exist in these five unhealthy behaviors that do not appear separately, and the interactions of these patterns are associated with SBs.

In this context, based on the exploratory LCA, we identified the potential patterns of unhealthy behaviors based on a large population of Chinese adolescents, and examined associations between the patterns of unhealthy behaviors and SBs. Also, this study may provide a new thought for suicide prevention and improve adolescents' mental health.

## Methods

### Data and Participants

This study used information from a large cross-sectional survey in China, which was approved by the Ethics Committee of Anhui Medical University (Approval Number 20140087). The data were collected from November 2015 to January 2016. In this study, 23,137 adolescents (aged 12–19 years) were enrolled from the junior school and the senior high school, located in six provinces in China, including both urban and rural regions, by using multistage stratified cluster sampling. Six provinces were selected by convenient sampling, including Shenyang (the capital of Liaoning province), Bengbu (northeastern of Anhui province), Yangjiang (southwestern coast of Guangdong province), Chongqing (one of China's four direct-controlled municipalities), Ulanchap (central inner Mongolia Autonomous Region), and Xinxiang (North of Henan province). Then, eight schools (two rural junior and two senior high schools, two urban junior, and two senior high schools) in each region were selected based on stratified cluster sampling. Lastly, four to six classes from each grade were randomly selected (grades 7–12) in each school, and all students in the selected classes were invited to the study, excluding participants who did not want to participate, who were absent on the survey day, and those having a history of psychiatric disorders or under treatment with psychiatric medication (participants with psychiatric disorders were primarily self-reported or reported by parents).

According to the principle of informed consent, the research staff explained the aim and process of the study to the students participating in the survey, and students were allowed to choose to participate or not. Students were required to complete the questionnaires during class time in about 20–30 min. The questionnaires were completed anonymously with unified instructions. A research staff was responsible for quality control of the questionnaires to answer the questions of the recipients and for collecting and proofreading the questionnaire. Excluding incomplete questionnaires (with more than 5% missing data), 22,628 valid questionnaires were received with an effective recovery rate of 97.8%. The mean age of the participants in this study was 15.36 (SD = 1.79) years. Of the 22,628 participants, 10,990 students were men (48.6%) and 11,638 were women (51.4%).

### Measures

Sociodemographic variables included sex (male or female), grade (junior or senior), place of residence (rural or urban), having any siblings (yes or no), residing at school or commuting to school, parental educational level (<High school degree or ≥High school degree), self-reported family economy (low, general, or high), and the number of friends (≤2, 3–5, or ≥6).

Suicidal behavior was assessed from three items (being suicidal ideation, suicide plan, and suicide attempt) according to the youth risk behavior surveillance system (YRBSS) (28). The details of the questions were as follows: (1) In the past 12 months, have you ever thought about killing yourself?; (2) In the past 12 months, have you ever made any plans to kill yourself?; (3) In the past 12 months, have you ever tried to kill yourself?. Response options of four-point Likert scale (none, once, 2–3 times, 4 times, or more than 4 times) are applied to each question. Students who responded as once or more times to any of the three questions were judged as having SB.

The definition of smoking and AU were based on YRBSS (28). Current smoking status was assessed by the item of “During the past 30 days, how many days did you smoke cigarettes?” as well as the current AU status. The options for both questions were as follows, “0” = 0 days, “1” = 1–9 days, “2” = 10–19 days, and “3” = 20–30 days. For these two items, 0 was chosen as no and the other options as yes (28). DPU was measured with the item “During the past 30 days, have you taken any diet pills or a liming tea without a doctor's advice to lose weight?,” with response options of 0 = 0 time; 1 = 1 time; 2 = 2–3 times; 3= 4 and over 4 times, in which option of 0 is considered as no and other options are considered as yes (29). The validity of these items was demonstrated in previous studies (30).

Problematic mobile phone use was assessed *via* the self-rating questionnaire for adolescent problematic mobile phone use (SQAPMPU), the standardized questionnaire for PMPU in adolescents (31). It consists of 13 items that respond to a four-point Likert scale (never, occasionally, sometimes, often, and always). Based on the prior literature, SQAPMPU global scores ≥28 were judged as PMPU (32–34). In this study, the Cronbach's alpha coefficient was 0.923.

Screen time was assessed by answers to the following questions, “On school days, how much time do you spend on average every day playing games or doing things unrelated to study on the computer?” And, too long ST was defined as ST >2 h/day according to the standard of the American Academy of Pediatrics and previous studies (35).

### Statistical Analysis

Latent classes of unhealthy behaviors were identified by LCA (Mplus version 7.4). In this study, we valued latent class models from one to five latent classes to ensure the most suitable number of latent classes. It is shown in a previous work that Akaike information criteria (AIC) and Bayesian information criteria (BIC) are the primary indices to select the latent class model (36). While BIC is a more reliable indicator for the model with fewer parameters or with a large sample size more than 1,000. Accordingly, adjusted BIC (aBIC) was used in this study *via* sample size calculation to determine the number of latent classes (32). The best suitable model is that both BIC and aBIC showed the lowest value (37). In addition, Lo–Mendell–Rubin Likelihood Ratio Test (LMR-LRT) and Bootstrapped likelihood ratio test (BLRT) were also used in this study, which are useful tools for class enumeration, indicating that the model of *k* classes is better than the model of *k–*1 classes when there is statistical significance (*p* < 0.05) (32, 37).

In addition, IBM SPSS version 23.0 was used for Chi-squared analysis and logistic regression analysis. Chi-squared tests were used to compare the prevalence of SBs among the different demographic variables. Multiple logistic regression analysis was used to ensure the relationships between unhealthy behaviors and SBs. And, this analysis was adjusted by sociodemographic characteristics. *p* < 0.05 was set as statistical significance.

## Results

### Descriptive Characteristics of the Samples

The prevalence of suicidal ideation, suicide plan, and suicide attempt was 14.1% (3,195), 7.3% (1,660), and 4.4% (1,000), respectively. Table 1 presented the occurrence of SBs according to frequency sociodemographic characteristics. In this study, for all three SBs, a higher rate was found in students of junior school, urban students, non-resident students, higher father's educational level, lower self-reported family economy, and fewer friends than correspondence groups (*p* < 0.05 for each). In addition, a higher rate of suicidal ideation was found in the female and only child. And for the suicide plan, the higher rate was in the students of only child and higher mother's educational level (*p* < 0.05 for each) (Table 1).

**Table 1:** Descriptive socio-demographic characteristics of the sample in the study, *n* (%).

**Variable**	**Total sample**	**Suicide ideation**	**Suicide plan**	**Suicide attempt**
Sex				
Male	10,990 (48.6)	1,430(13.0)	826 (7.5)	512 (4.7)
Female	11,638 (51.4)	1,765(15.2)	834 (7.2)	488 (4.2)
*χ*^2^		21.63***	1.02	2.90
Grade				
Junior school	11,993 (53.0)	1,809 (15.1)	963 (8.0)	586 (4.9)
Senior high school	10,635 (47.0)	1,386 (13.0)	697 (6.6)	414 (3.9)
*χ*^2^		19.56***	18.06***	13.17***
Place of residence				
Rural	10,882 (48.1)	1,460 (13.4)	758 (7.0)	449 (4.1)
Urban	11,746 (51.9)	1,735 (14.8)	902 (7.7)	551 (4.7)
*χ*^2^		8.54**	4.23*	4.27*
Having any siblings				
No	9,720 (43.0)	1,458 (15.0)	772 (7.9)	448 (4.6)
Yes	12,908 (57.0)	1,737 (13.5)	888 (6.9)	552 (4.3)
*χ*^2^		10.89**	9.22**	1.45
Resident students				
Yes	11,320 (50.0)	1,525 (13.5)	758 (6.7)	414 (3.7)
No	11,308 (50.0)	1,670 (14.8)	902 (8.0)	586 (5.2)
*χ*^2^		7.84**	13.65***	31.14***
Father's educational level^a^				
<High school degree	13,006 (57.5)	1,708 (13.1)	860 (6.6)	525 (4.0)
≥High school degree	9,424 (41.6)	1,427 (15.1)	762 (8.1)	449 (4.8)
*χ*^2^		18.36***	12.64***	6.97**
Mother's educational level^b^				
<High school degree	14,335 (63.4)	1,938 (13.5)	973 (6.8)	606 (4.2)
≥High school degree	8,105 (35.8)	1,207 (14.9)	654 (8.1)	375 (4.6)
*χ*^2^		8.10	12.64***	1.96
Self-reported family economy				
Bad	3,240 (14.3)	622 (19.2)	347 (10.7)	196 (6.1)
General	16,345 (72.2)	2,090 (12.8)	1,035 (6.3)	623 (3.8)
Good	3,043 (13.4)	483 (15.8)	278 (9.1)	181 (6.0)
*χ*^2^		100.55***	92.98***	51.51***
Number of close friends				
≤2	5,514 (24.4)	1,017 (18.4)	560 (10.2)	324 (5.9)
3~5	9,620 (42.5)	1,225 (12.7)	580 (6.0)	345 (3.6)
≥6	7,494 (33.1)	953 (12.7)	520 (6.9)	331 (4.4)
*χ*^2^		112.43***	90.41***	43.50***

a *198 students have no information about their father.*

b *188 students have no information about their mother;*

* *p < 0.05;*

** *p < 0.01;*

*** *p < 0.001*.

### Identification of Latent Class

The rates of smoking, AU, DPU, PMPU, and ST were found to be a total of 2.8% (635), 16.8% (3,799), 4.8% (1,086), 25.4% (5,752), and 16.3% (3,690) among Chinese adolescents, respectively. According to LCA, the patterns of unhealthy behaviors were identified. As shown in Table 2, the five-class model did not report the best log-likelihood value and the *p*-value of LMR-LRT and BLRT both was not statistically significant, thus it was not considered further. Both BIC (77,654.647) and aBIC (77,581.554) were the lowest in the four-class model, where LMR-LRT and BLRT were statistically significant (*p* < 0.001). Although, *p-*value of the LMR-LRT and BLRT test was also significant for 2- and 3-class models, the 4-class model showed the lowest AIC, BIC, and aBIC values. These findings indicated the better fit of this 4-class model than other models in the present study. Accordingly, we utilized the 4-class model and thus identified four latent classes (Table 2).

**Table 2:** Model fit statistics for each of the fitted latent class analysis (LCA) models.

**Statistic**	***df***	**AIC**	**BIC**	**aBIC**	**LMR-LRT**	**BLRT**	**Entropy**
2 Classes	11	77,820.87	77,909.17	77,874.21	<0.0001	<0.0001	0.581
3 Classes	17	77,531.15	77,667.6	77,613.58	<0.0001	<0.0001	0.78
4 Classes	23	77,470.03	77,654.65	77,581.55	<0.0001	<0.0001	0.728
5 Classes	29	77,473.07	77,705.85	77,613.69	0.0822	0.1935	0.717

*df, degrees of freedom; AIC, Akaike information criteria; BIC, Bayesian information criteria; LMR-LRT, Lo–Mendell–Rubin Likelihood Ratio Test; BLRT, Bootstrapped Likelihood Ratio Tests*.

**Figure 1:**
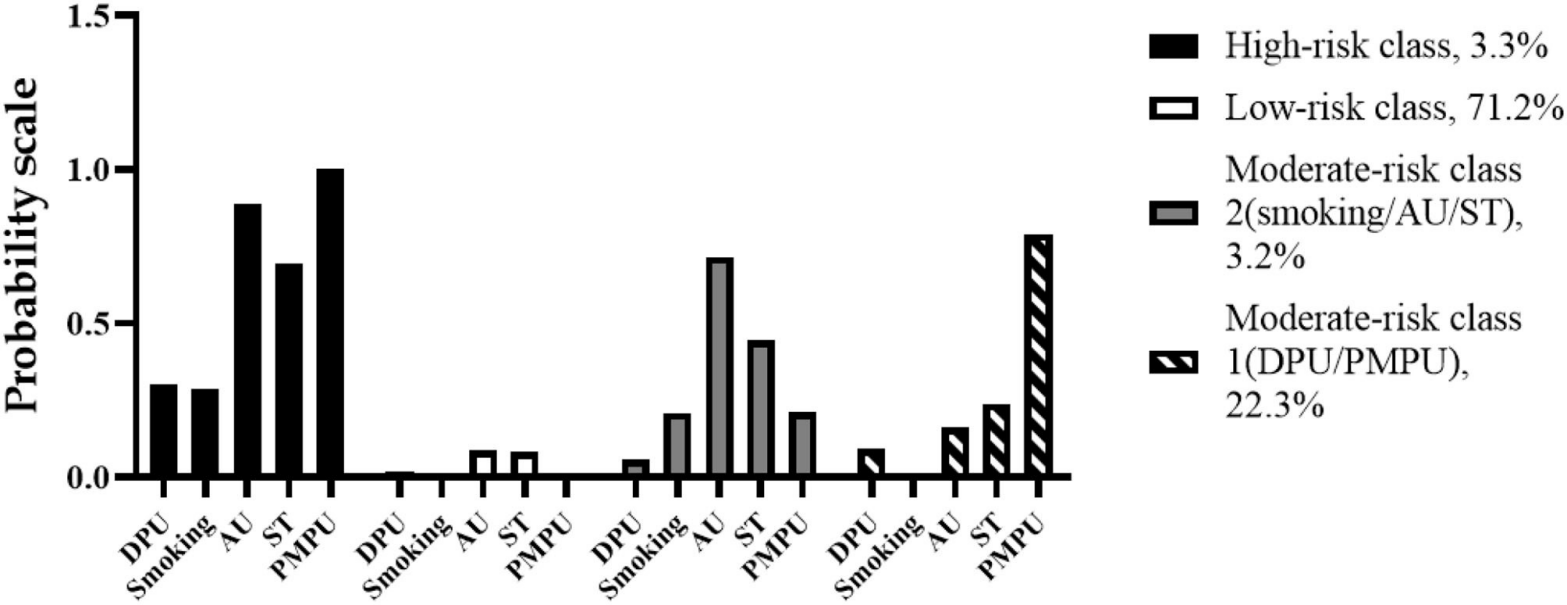
The estimated probabilities of five behaviors among the four patterns.

As shown in Figure 1, latent class 1 was recognized as the low-risk class, which was composed of 16,113 (71.2%) of the samples. In this pattern, none of the participants reported PMPU (0%), and the participants simultaneously reported the lowest rate of the other four unhealthy behaviors. In contrast, latent class 4 was the high-risk class that included 3.3% (747) of the samples. Majority of the students in this pattern reported smoking (28.8%), AU (88.6%), DPU (30.1%), ST (69.6%), and all of them were engaged in PMPU (100%). The other two latent classes were termed moderate-risk classes 1 and 2, which contained 22.3% (5,055) and 3.2% (713) of the samples, respectively. Moderate-risk class 1 (latent class 2) showed high reporting rates on DPU (9.1%) and PMPU (79.1%) compared to moderate-risk class 2, which was thus defined as “DPU and PMPU class.” In contrast, moderate-risk class 2 (latent class 3) showed high reporting rates on smoking (20.9%), AU (71.6%), and ST (44.5%), which was thus defined as “smoking, AU, and ST class” (Figure 1).

### The Characteristics of Participants With Different Patterns of Unhealthy Behaviors

Table 3 presented the sociodemographic characteristics of participants with different patterns of unhealthy behaviors. Female students were more significantly prone to be classified into the low-risk class, while the same tendency was found in students of junior school, with siblings, residents, and lower fathers' educational level (*p* < 0.05 for each). Also, respondents who reported general family economy were more likely to cluster into the low-risk class (*p* < 0.05). However, students with fewer close friends were less likely to be in the low-risk class (*p* < 0.05) (Table 3).

**Table 3:** Description of students with patterns of unhealthy behaviors in Chinese adolescents.

**Variable**	**Total sample**	**Low-risk class**	**Moderate-risk class 1 (DPU /PMPU)**	**Moderate-risk class 2 (smoking/AU/ST)**	**High-risk class (smoking/AU/DPU/PMPU/ST)**	***χ* ^2^**
Sex						287.47***
Male	10,990 (48.6)	7,528 (68.5)	2,449 (22.3)	528 (4.8)	485 (4.4)	
Female	11,638 (51.4)	8,585 (73.8)	2,606 (22.4)	185 (1.6)	262 (2.3)	
Grade						103.90***
Junior school	11,993 (53.0)	8,871 (74.0)	2,396 (20.0)	330 (2.8)	396 (3.3)	
Senior high school	10,635 (47.0)	7,242 (68.1)	2,659 (25.0)	383 (3.6)	351 (3.3)	
Place of residence						5.96
Rural	10,882 (48.1)	7,796 (71.6)	2,425 (22.3)	331 (3.0)	330 (3.0)	
Urban	11,746 (51.9)	8,317 (70.8)	2,630 (22.4)	382 (3.3)	417 (3.6)	
Having any siblings						9.96*
No	9,720 (43.0)	6,840 (70.4)	2,220 (22.8)	305 (3.1)	355 (3.7)	
Yes	12,908 (57.0)	9,273 (71.8)	2,835 (22.0)	408 (3.2)	392 (3.0)	
Resident students						95.20***
Yes	11,320 (50.0)	7,869 (69.5)	2,806 (24.8)	337 (3.0)	308 (2.7)	
No	11308 (50.0)	8244 (72.9)	2249 (19.9)	376 (3.3)	439 (3.9)	
Father's educational level^a^						12.03**
<High school degree	13,006 (57.5)	9,283 (71.4)	2,945 (22.6)	398 (3.1)	380 (2.9)	
≥High school degree	9,424 (41.6)	6,712 (71.2)	2,062 (21.9)	301 (3.2)	349 (3.7)	
Mother's educational level^b^						6.50
<High school degree	14,335 (63.4)	10,204 (71.2)	3,240 (22.6)	456 (3.2)	435 (3.0)	
≥High school degree	8,105 (35.8)	5,801 (71.6)	1,773 (21.9)	242 (3.0)	289 (3.6)	
Self-reported family economy						135.77***
Bad	3,240 (14.3)	2,102 (64.9)	880 (27.2)	134 (4.1)	124 (3.8)	
General	16,345 (72.2)	11,920 (72.9)	3,502 (21.4)	457 (2.8)	466 (2.9)	
Good	3,043 (13.4)	2,091 (68.7)	673 (22.1)	122 (4.0)	157 (5.2)	
Number of close friends						90.98***
≤2	5,514 (24.4)	3,788 (68.7)	1,392 (25.2)	148 (2.7)	186 (3.4)	
3~5	9,620 (42.5)	6,895 (71.7)	2,187 (22.7)	279 (2.9)	259 (2.7)	
≥6	7,494 (33.1)	5,430 (72.5)	1,476 (19.7)	286 (3.8)	302 (4.0)	

a *198 students have no information about their father.*

b *188 students have no information about their mother;*

* *p < 0.05;*

** *p < 0.01;*

*** *p < 0.001*.

### Multiple Logistic Regression Analysis

Table 4 showed the associations between the four patterns of unhealthy behaviors and SBs. After adjusting for sociodemographic characteristics, compared with the low-risk class, all other patterns were seen to be positively related to SBs. Concerning suicide ideation, the adjusted odds ratio [OR; 95% confidence interval (CI)] was 3.30 (3.03–3.59), 2.35 (1.91–2.88), and 6.08 (5.16–7.16) for moderate-risk class 1, moderate-risk class 2, and high-risk class, respectively. Compared with the low-risk class, the results of the suicide plan suggested the values of OR (95% CI) of moderate-risk class 1, moderate-risk class 2, and high-risk class were 3.92 (3.50–4.39), 3.20 (2.49–4.11), and 7.96 (6.58–9.61), respectively. Moreover, compared with the low-risk class, the others patterns had higher risks of suicide attempt (OR_high−riskclass_ = 12.36, 95% CI: 9.95–15.35; OR_moderate−riskclass1_ = 5.59, 95% CI: 4.81–6.49; OR_moderate−riskclass2_ = 4.17, 95% CI: 3.07–5.66) (Table 4).

**Table 4:** Associations of SBs and patterns of unhealthy behaviors in Chinese adolescents.

**Latent class of unhealthy behaviors**	**Suicide ideation**		**Suicide plan**		**Suicide attempt**	
	***n* (%)**	**Adjusted OR (95% CI)**	***n* (%)**	**Adjusted OR (95% CI)**	***n* (%)**	**Adjusted OR (95% CI)**
High-risk class (smoking/AU/DPU/PMPU/ST)	285 (38.2)	6.08 (5.16~7.16)***	197(26.4)	7.96 (6.58–9.61)***	153 (20.5)	12.36 (9.95~15.35)***
Moderate-risk class 1 (DPU /PMPU)	1,280 (25.3)	3.30 (3.03~3.59)***	725 (14.3)	3.92 (3.50–4.39)***	487 (9.6)	5.59 (4.81~6.49)***
Moderate-risk class 2 (smoking/AU/ST)	126 (17.7)	2.35 (1.91~2.88)***	82 (11.5)	3.20 (2.49–4.11)***	54 (7.6)	4.17 (3.07~5.66)***
Low-risk class	1,504 (9.3)	Ref.	656 (4.1)	Ref	306 (1.9)	Ref.

*OR, odds ratio; CI, confidence interval; Adjusted OR, adjusted for sex, grade, place of residence, having any siblings, resident student, educational level of father and mother, self-reported family economy, and number of friends;*

*** *p < 0.001 compared with reference*.

## Discussion

In this study, we found that SBs are a common phenomenon in adolescents in China. Moreover, we found that female students showed more suicidal ideation (15.17%) than male students (13.01%), which is consistent with other reports (4, 38–40). Usually, female students are more prone to have suicidal ideation because of their predisposition to depression (40). Meanwhile, some previous studies showed that female students had more socioeconomic pressure, mental disorders, school, and violence, so they were more prone to suffer a suicide attempt (41). However, in this study, there was no apparent difference between the suicide plan and the suicide attempt between male and female students. This difference may be caused by the different samples, different scales, and different cultures of participants. Furthermore, in this study, we found that SBs were more in students of junior students, only child, urban students, non-resident students, higher parental educational level, low self-reported family economy, and fewer friends than the correspondence groups. And, this result is partly consistent with previous studies (6, 42).

In the present study, we focus on different patterns of unhealthy behaviors and examine the associations between latent classes of unhealthy behaviors and SBs in a large Chinese sample. This modeling helps to link a broad range of factors including smoking, AU, DPU, PMPU, and ST behaviors. In this study, we identified four patterns of unhealthy behaviors, which differ from previous research examining multiple unhealthy behaviors among community participants (25), possibly because the types of unhealthy behaviors are not completely consistent and the age range or country of the participants are different. Intriguingly, unhealthy behaviors appeared to show two different clustering patterns in the moderate-risk classes. The moderate-risk class 1 (DPU/PMPU) had high rates of DPU and PMPU. Though previous studies did not show the relationship between DPU and PMPU directly, both unhealthy weight control behaviors (including DPU) and PMPU were reported to be related to adolescent mental health including SBs (43–45). Usually, mobile phone use is related to screen media use, but in this study, ST is not clustered with PMPU, which may need further investigations to elucidate. In moderate-risk class 2 (smoking/AU/ST), students reported higher rates of smoking, AU, and ST. It is commonly known smoking and AU usually coexist, which collaboratively influence the health of adolescents (40, 46). And the present study showed that adolescents who prefer smoking and AU are also more likely to have ST for a long time, which is consistent with previous studies (47). In addition, our results showed that the proportion of the high-risk class (smoking/AU/DPU/PMPU/ST) was only 3.3%. However, given China's population base, it is necessary to identify and intervene in high-risk class (smoking/AU/DPU/PMPU/ST).

Our results indicated that men, senior school students, only children, commuting students, student with higher parental educational level, and less close friends were more engaged in patterns with unhealthy behaviors, especially in moderate-risk class 2 (smoking/AU/ST) and high-risk class (smoking/AU/DPU/PMPU/ST), which was consistent with previous studies (24, 32). This means that it may be more efficient to take into account demographic heterogeneity in identifying patterns of unhealthy behaviors. Intriguingly, this study found that students with general family economics were more likely to be in the low-risk class, whereas students with reports of bad and good family economics were more likely to be classified into moderate-risk class 1, moderate-risk class 2, and high-risk class. The reason for this may be that students with general family economics have higher health literacy, which is a protective factor for unhealthy behaviors (48).

Our study further showed that students who engaged in more unhealthy behaviors always exhibited a higher risk of SBs. Compared to the low-risk class, all three subclasses (the high-risk class, moderate-risk class 1, and moderate-risk class 2) tend to have higher rates of SBs. This implied that certain unhealthy behaviors usually occurred in simultaneously, concurrently, and mutually affecting SBs, and adolescents exposed to similar conditions may show different outcomes because of the heterogeneity of unhealthy behaviors. Therefore, different strategies should be developed to target different groups of adolescents to prevent and control unhealthy behaviors and SBs. For the general population, such as students in the low-risk class, a series of courses on physical activity and health education to improve their health literacy may be effective (32, 49). Nevertheless, for the high-risk population, mindfulness-based interventions may be appropriate and necessary (50–52). Furthermore, the sense of weight control for adolescents was explained by the Social Cognitive Theory of imitating behaviors from role models, and under this condition, cognitive behavioral therapy may be useful to prevent unhealthy weight loss due to DPU (53, 54). In addition, physical activity is a significant way to reduce problematic smartphone use among adolescents (55). Therefore, cognitive behavioral therapy with physical activity may be an appropriate way in moderate-risk class 1. Moreover, adolescents usually receive cigarettes and alcohol first from their friends, therefore group therapy, namely, peer education and development of adolescents' skills to say “no,” will be an effective preventive strategy (56). Moreover, a previous study indicated that autonomous motivation acts as a significant mediator of changes in ST (57). Taken together, it may be an effective intervention for moderate-risk class 2 when using group therapy through peer education and autonomous motivation. In summary, as unhealthy behaviors occur simultaneously and cluster together rather than appearing separately, it is essential to consider the characteristics of different patterns to develop preventive strategies and prepare a suitable invention plan.

In this study, we use a relatively large sample size, which will better reflect the situation of the whole population. Furthermore, variables of unhealthy behaviors used in this study cover not only substance use behaviors, but also non-substance use behaviors. More importantly, we utilized the LCA method to illustrate the association between unhealthy behaviors and SBs, which will provide more reasonable and useful information for further establishing prevention programs, than single-factor analysis. All these will increase the reliability and applicability of this study. However, there are also some limitations in the present study. Firstly, we used self-reported data, which resulted in possibly inevitable recall and reporting bias. Meanwhile, a cross-sectional design could not imply causal relationships between unhealthy behaviors and SBs. Longitudinal studies should be adopted to verify causal relationships in follow-up studies. Moreover, it should be noted that, in addition to the unhealthy behaviors investigated in this study, other unhealthy behaviors (e.g., excessive sugar intake and insufficient physical activity) as well as the psychological factor regarding suicide (such as anxiety, depression, and affective temperament types) may also be of critical importance, as the major unhealthy behaviors may vary in different samples and occasions. Therefore, further studies and considerations are warranted to focus on the different types of unhealthy behaviors and cluster populations according to the major unhealthy behaviors, for the development of the appropriate preventive strategies.

## Conclusion

Taken together, similar to many previous studies, we report here that SBs have a high prevalence rate among adolescents. By focusing on unhealthy behaviors, we, through LCA analysis, found that unhealthy behaviors do not act independently on SBs, but act synergistically, which may be related to the interrelated characteristics of unhealthy behaviors. Namely, if we want to prevent SBs by simply changing a certain kind of unhealthy behaviors, the result may be unsatisfactory. Therefore, compared with intervening some unhealthy behaviors alone, more benefits can be obtained by intervening multiple unhealthy behaviors in a specific group (high-risk class, moderate-risk class 1 and moderate-risk class 2). Many studies indicated that the improvement of mindfulness-based intervention, group therapy, and physical activity could effectively intervene in adolescents on unhealthy risk behaviors. We thus anticipated that SBs in adolescents could be effectively prevented by identifying groups with unhealthy behaviors and developing effective methods targeting these groups of unhealthy behaviors.

## Data Availability Statement

The original contributions presented in the study are included in the article/supplementary material, further inquiries can be directed to the corresponding authors.

## Ethics Statement

The studies involving human participants were reviewed and approved by the Ethics Committee of Anhui Medical University (NO. 20140087). Written informed consent to participate in this study was provided by the participants' legal guardian/next of kin.

## Author Contributions

SZ and JF were responsible for the conception, provided funding for the project and design of the study. CG, YX, YC, ZX, JH, and XH were involved in data collection. SZ and YW were responsible for subjects' recruitment and data collection. CG and YX conducted the statistical analysis. CG and YX wrote the first draft of the paper, which was critically revised by SZ and JF. All authors contributed to interpretation of the findings. All authors contributed to the article and approved the submitted version.

## Funding

This work was supported by the Innovation Team Project of Anhui Medical College (WJH2022001t), the National Ministry of Education Humanities and Social Science Research Planning Fund Project (21YJAZH120), the Natural Science Foundation in Higher Education of Anhui (KJ2020A0209), and the National Natural Science Foundation of China (81402699 and 81573512). The funders had no role in study design, data collection and analysis, decision to publish, or preparation of the manuscript.

## Conflict of Interest

The authors declare that the research was conducted in the absence of any commercial or financial relationships that could be construed as a potential conflict of interest.

## Publisher's Note

All claims expressed in this article are solely those of the authors and do not necessarily represent those of their affiliated organizations, or those of the publisher, the editors and the reviewers. Any product that may be evaluated in this article, or claim that may be made by its manufacturer, is not guaranteed or endorsed by the publisher.

## Abbreviations

SBs: Suicidal behaviors
AU: Alcohol use
ST: Screen time
PMPU: Problematic mobile phone use
DPU: Diet pill use
LCA: Latent class analysis
AIC: Akaike information criteria
BIC: Bayesian information criteria
LMR-LRT: Lo–Mendell–Rubin Likelihood Ratio Test)
BLRT: Bootstrapped Likelihood Ratio Test
*Df*: degrees of freedom
OR: odds ratio
CI: confidence interval.
